# Beyond Type 1 Diabetes: Autoimmune Gastritis-Driven Multisystem Disease in Autoimmune Polyglandular Syndrome Type 3

**DOI:** 10.7759/cureus.104463

**Published:** 2026-02-28

**Authors:** Niyas Khalid Ottu Para, Roa A Ahmmed, Malaz Mohamed, Seema Rab

**Affiliations:** 1 Internal Medicine, Burjeel Hospital, Abu Dhabi, ARE

**Keywords:** autoimmune atrophic gastritis, autoimmune hypothyroidism, autoimmune polyglandular syndrome type 3, pernicious anemia, type 1 diabetes (t1d)

## Abstract

Type 1 diabetes mellitus (T1DM) is frequently managed as an isolated endocrine disorder despite being a sentinel diagnosis for broader autoimmune clustering. Autoimmune polyglandular syndrome type 3 (APS-3), defined by the coexistence of T1DM with autoimmune thyroid disease and/or autoimmune gastritis in the absence of adrenal insufficiency, remains underrecognized in routine clinical practice, particularly when manifestations extend beyond classical endocrine organs. We report a diagnostically delayed case of APS-3 in a middle-aged man whose long-standing gastrointestinal symptoms, nutritional deficiencies, and pancreatic dysfunction were repeatedly attributed to complications of diabetes rather than a unifying autoimmune process. The patient was ultimately found to have autoimmune atrophic gastritis with pernicious anemia, extreme hypergastrinemia, autoimmune hypothyroidism, and severe exocrine pancreatic insufficiency with radiologic pancreatic atrophy. Subsequent episodic biliary obstruction further complicated the diagnostic course but ultimately reinforced the need for an integrative approach. This case highlights critical gaps in endocrine-centric diagnostic frameworks, illustrates how autoimmune gastritis and pancreatic involvement can dominate the clinical picture, and underscores the importance of symptom-driven expansion of autoimmune surveillance in patients with T1DM to prevent prolonged morbidity.

## Introduction

Type 1 diabetes mellitus (T1DM) is among the most common organ-specific autoimmune diseases and often represents the earliest clinical manifestation of systemic autoimmunity [1]. Autoimmune polyglandular syndromes describe defined constellations of autoimmune disorders affecting endocrine and non-endocrine organs; however, their recognition in everyday clinical practice remains inconsistent [2-4]. Autoimmune polyglandular syndrome type 3 (APS-3), characterized by T1DM in association with autoimmune thyroid disease and/or autoimmune gastritis in the absence of adrenal insufficiency, is particularly prone to underdiagnosis because its manifestations frequently fall outside traditional endocrine algorithms [3,4].

Autoimmune polyglandular syndromes are traditionally classified into three major subtypes. APS-1 is a rare monogenic disorder presenting in childhood with chronic mucocutaneous candidiasis, hypoparathyroidism, and adrenal insufficiency. APS-2 is characterized by autoimmune adrenal insufficiency in combination with autoimmune thyroid disease and/or T1DM. In contrast, APS-3 involves T1DM with autoimmune thyroid disease and/or autoimmune gastritis in the absence of adrenal failure and typically follows a more indolent, heterogeneous course.

In routine practice, follow-up of patients with T1DM prioritizes glycemic control and surveillance for thyroid or adrenal dysfunction, while gastrointestinal symptoms, nutritional deficiencies, and systemic complaints are often attributed to diabetic complications or managed symptomatically. Autoimmune gastritis, despite its well-established association with T1DM, is frequently recognized late, after years of iron deficiency, vitamin B12 deficiency, and gastrointestinal morbidity [5].

Autoimmune gastritis is characterized by immune-mediated destruction of gastric parietal cells, leading to hypochlorhydria, intrinsic factor deficiency, and subsequent iron and vitamin B12 malabsorption. Its prolonged subclinical course often results in delayed recognition despite progressive biochemical abnormalities.

Similarly, pancreatic involvement in autoimmune disease is commonly conceptualized solely in terms of β-cell destruction, with exocrine pancreatic dysfunction remaining underappreciated [6-8]. Gastrointestinal symptoms in T1DM are frequently attributed to diabetic autonomic neuropathy or gastroparesis. However, structural pancreatic atrophy and exocrine insufficiency represent distinct pathophysiologic entities that require separate diagnostic consideration.

We present a case of APS-3 in which prolonged patient suffering resulted from fragmented, organ-specific care and misattribution of symptoms to complicated diabetes. This case illustrates how autoimmune gastritis and pancreatic exocrine insufficiency can drive the clinical presentation, exposes important pitfalls in current endocrine practice, and emphasizes the need for a more integrative, symptom-driven approach to autoimmune disease recognition in patients with T1DM [3,4].

## Case presentation

A 37-year-old man with a 29-year history of T1DM (diagnosed in 1997) and managed with intensive insulin therapy was referred for evaluation of chronic gastrointestinal symptoms and progressive functional decline. For more than five years, he had experienced persistent epigastric discomfort, bloating, early satiety, intermittent loose stools, and postprandial fullness, accompanied by fatigue, reduced exercise tolerance, and fluctuating weight. During this period, he had been treated intermittently with proton pump inhibitors for approximately four to five years, with only partial and temporary symptomatic relief. His gastrointestinal complaints and constitutional symptoms were largely attributed to long-standing diabetes and presumed diabetic complications, and no unifying autoimmune evaluation had been undertaken during this time.

On clinical examination, the patient appeared chronically fatigued but hemodynamically stable. Abdominal examination revealed mild epigastric tenderness without guarding or organomegaly. There were no features of adrenal insufficiency or overt malnutrition. Neurologic examination demonstrated no focal deficits.

Initial laboratory findings

Routine hematologic investigations, as shown in Table 1, demonstrated chronic mild anemia, with hemoglobin values ranging between 10.9-11.8 g/dL, hematocrit 28-37%, and mean corpuscular volume in the low-normal range. Iron studies revealed low serum iron levels with preserved ferritin concentrations, suggesting iron-restricted erythropoiesis rather than isolated iron deficiency. Vitamin B12 levels were persistently low despite intermittent supplementation. Homocysteine was elevated at 19 µmol/L, supporting functional vitamin B12 deficiency. Folate levels were normal to elevated. Biochemical evaluation demonstrated suboptimal glycemic control, with hemoglobin A1c values ranging from 8.1% to 8.8%. Additional abnormalities included hypertriglyceridemia, low high-density lipoprotein cholesterol, and elevated C-reactive protein. Renal and hepatic function tests were initially preserved.

**Table 1:** Summary of laboratory investigations. Summary of hematological, inflammatory, hepatic, metabolic, coagulation, lipid, renal, and micronutrient laboratory parameters recorded during the patient’s hospitalization. Where two values are presented separated by a dash (–), these represent the range of values observed between admission and subsequent measurements during the clinical course. Values connected by an arrow (→) indicate sequential measurements demonstrating temporal trends. All values are shown alongside their respective reference ranges. WBC: white blood cells; RBC: red blood cells; CRP: C-reactive protein; ALT (SGPT): alanine aminotransferase; AST (SGOT): aspartate aminotransferase; ALP: alkaline phosphatase; GGT: gamma-glutamyl transferase; HbA1c: glycated hemoglobin; PT: prothrombin time; APTT: activated partial thromboplastin time; LDL: low-density lipoprotein; HDL: high-density lipoprotein

Laboratory parameter	Findings	Reference range
WBC	11.23–12.40 × 10⁹/L	4–10 × 10⁹/L
RBC	3.6–4.4 × 10¹²/L	4.5–5.9 × 10¹²/L
Hemoglobin	9.5–12.0 g/dL	13–17 g/dL
Hematocrit	28–37%	40–50%
Platelets	468–499 × 10⁹/L	150–400 × 10⁹/L
Neutrophils	Up to 85%	40–75%
Lymphocytes	10–17%	20–45%
CRP	5.2 → 10.5 mg/L	<5 mg/L
ALT (SGPT)	204 → 631 U/L	<45 U/L
AST (SGOT)	230 → 545 U/L	<40 U/L
ALP	193 → 322 U/L	40–129 U/L
GGT	257 → 648 U/L	<60 U/L
Total protein	56–62 g/L	64–83 g/L
Albumin	34–36 g/L	35–50 g/L
HbA1c	8.1–8.8%	<5.7%
Urine letones	+4	Negative
PT	9.1–9.7 seconds	11–13.5 seconds
APTT	22–24 seconds	25–35 seconds
Triglycerides	4.6–6.5 mmol/L	<1.7 mmol/L
LDL cholesterol	5.6 mmol/L	<3.0 mmol/L
HDL cholesterol	0.77 mmol/L	>1.0 mmol/L
Creatinine	100 µmol/L	64–104 µmol/L
Serum iron	42 µg/dL	60–170 µg/dL
Ferritin	65 ng/mL	30–400 ng/mL
Vitamin B12	125–181 pg/mL	200–900 pg/mL
Folate	45.5 ng/mL	>4 ng/mL

Autoimmune evaluation

Given the persistence of vitamin B12 deficiency and anemia, an autoimmune workup was pursued, as shown in Table 2. Intrinsic factor IgG antibodies were strongly positive (54 U/mL), and parietal cell antibodies were strongly positive. Fasting serum gastrin was markedly elevated at 2,423 pg/mL (reference range: 13-115 pg/mL), consistent with profound hypochlorhydria due to corpus-predominant gastric atrophy.

**Table 2:** Autoimmune, thyroid, and gastrointestinal serological profile. Summary of autoimmune gastric antibodies, thyroid function tests, celiac serology, systemic autoimmune markers, and renin-aldosterone profile with corresponding reference ranges. Abnormal values are indicated where applicable. TSH: thyroid-stimulating hormone; FT4: free thyroxine; FT3: free triiodothyronine; TPO: thyroid peroxidase; TTG: tissue transglutaminase; ANA: antinuclear antibody

Parameter	Value	Reference range
Intrinsic factor antibody (IgG)	54.0 U/mL (positive)	Negative <6.0 U/mL
Parietal cell antibody	Strong positive	Negative
Gastrin	2,423 pg/mL	13–115 pg/mL
TSH	22.5 mIU/L	0.4–4.0 mIU/L
FT4	4.59 pmol/L	12–22 pmol/L
FT3	3.35 pmol/L	3.1–6.8 pmol/L
TPO antibody	600 IU/mL	<35 IU/mL
TTG IgM	Negative	Negative
TTG IgG	Negative	Negative
ANA	Negative	Negative
Anti-dsDNA	Negative (<0.5)	<1.0
Renin	Normal	4.4–46.1 mIU/L
Aldosterone	Normal	30–355 pmol/L

Autoimmune atrophic gastritis was therefore established by the combination of intrinsic factor and parietal cell autoantibody positivity in conjunction with extreme hypergastrinemia. The constellation of autoantibody positivity, biochemical vitamin B12 deficiency, iron-restricted erythropoiesis, and marked hypergastrinemia established pernicious anemia as a manifestation of corpus-predominant autoimmune gastric destruction. *Helicobacter pylori* stool antigen testing was negative, reducing the likelihood of secondary infectious gastritis as an alternative etiology. The patient reported having undergone prior upper gastrointestinal endoscopy in his home country; however, no endoscopic or histopathologic documentation was available for review at the time of evaluation.

Screening for additional autoimmune disease demonstrated evolving primary hypothyroidism, with persistently elevated thyroid-stimulating hormone levels and borderline-to-low free thyroxine levels. Celiac disease screening using tissue transglutaminase IgA and IgG antibodies was negative. Antinuclear antibody and anti-double-stranded DNA testing were negative. Morning cortisol and adrenocorticotropic hormone levels were within normal limits, excluding adrenal insufficiency and supporting classification as APS-3 rather than APS-2. Renin and aldosterone levels were also within normal limits.

Pancreatic and gastrointestinal assessment

Despite correction attempts for vitamin B12 deficiency, gastrointestinal symptoms persisted. Exocrine pancreatic function testing revealed profoundly reduced fecal elastase levels, diagnostic of severe exocrine pancreatic insufficiency. Fecal calprotectin was mildly elevated, suggesting low-grade intestinal inflammation. Serum IgG subclass analysis demonstrated normal total IgG with selective reduction of the IgG1 subclass; IgG4 levels were normal, arguing against IgG4-related disease. Serum lipase and amylase levels were not elevated. Carbohydrate antigen 19-9 levels were within normal limits. Table 3 summarizes the pancreatic biochemical profile, exocrine pancreatic function assessment, tumor marker evaluation, and immunoglobulin subclass analysis obtained during the patient’s clinical course.

**Table 3:** Pancreatic enzymes, exocrine function, tumor marker, and IgG subclass profile. Summary of pancreatic enzyme trends, fecal elastase as a marker of exocrine pancreatic function, tumor marker status, and serum total IgG with IgG subclass distribution. Values are presented with corresponding reference ranges. Sequential amylase and lipase values (105 → 114 → 142 U/L and 218 → 64 → 479 U/L, respectively) represent serial measurements obtained at different time points during hospitalization, reflecting dynamic biochemical changes over the clinical course. Abnormal findings are indicated where applicable. IgG: immunoglobulin G; CA 19-9: carbohydrate antigen 19-9

Parameter	Value	Reference range
Fecal elastase	1.15 µg/g (very low)	>200 µg/g stool
Total IgG	Normal	7–16 g/L
IgG subclass 1	3.57 g/L (low)	4.9–11.4 g/L
IgG subclass 2	Normal	1.5–6.4 g/L
IgG subclass 3	Normal	0.2–1.1 g/L
IgG subclass 4	Normal	0.08–1.4 g/L
Amylase	105 → 114 → 142 U/L (high trend)	28–100 U/L
Lipase	218 → 64 → 479 U/L (fluctuating/high)	13–60 U/L
CA 19-9	Normal	<37 U/mL

Radiologic findings

**Figure 1:**
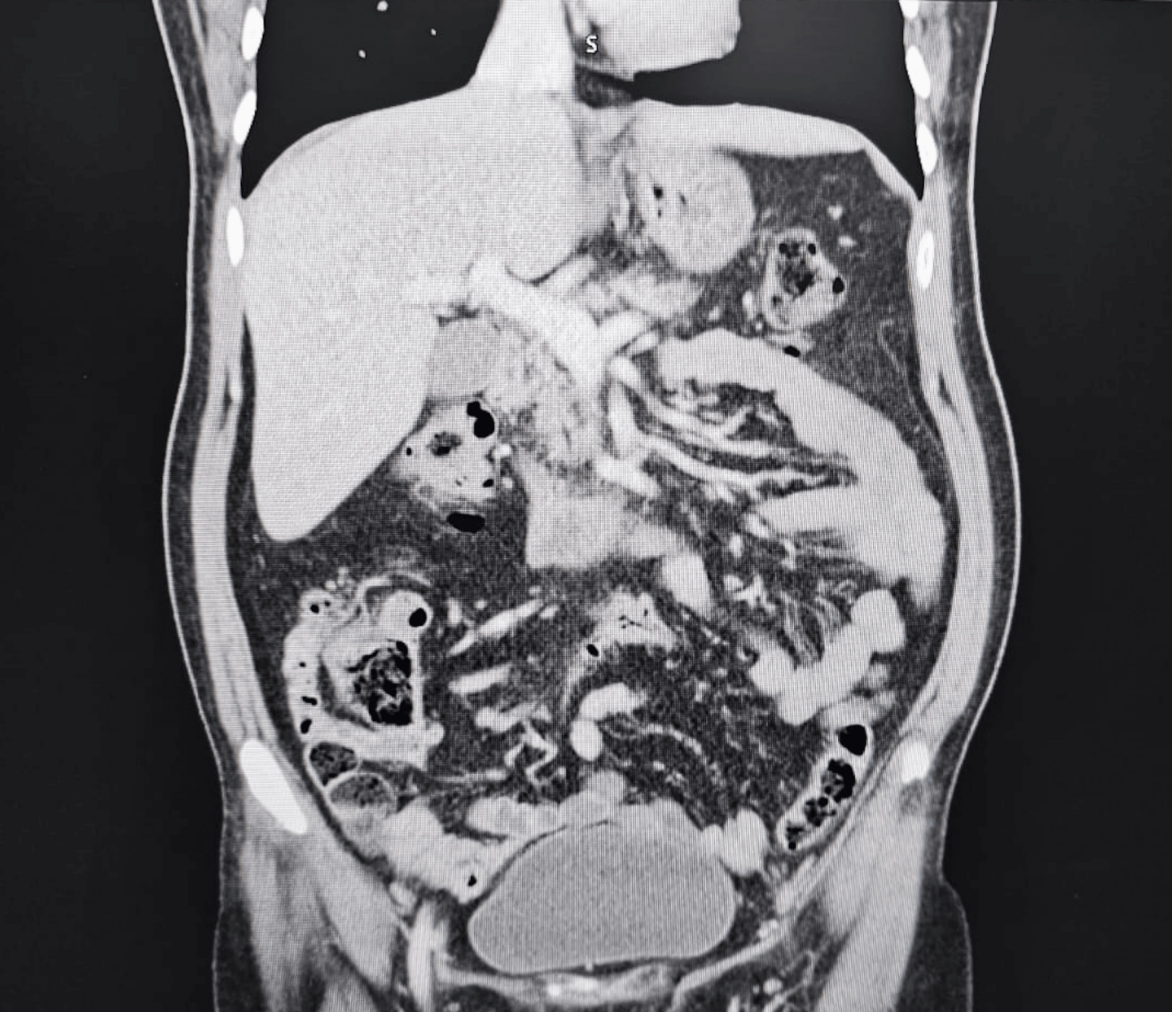
CT of the abdomen showing features of chronic pancreatitis with main pancreatic duct dilatation and head calcification. Contrast-enhanced axial CT image of the abdomen showing diffuse pancreatic parenchymal atrophy and peripancreatic fat stranding. A punctate calcification is visualized in the pancreatic head along the main pancreatic duct. The duct is markedly dilated (≈9 mm) proximally with abrupt distal narrowing at the ampullary region and subtle peripheral wall enhancement. No evidence of pancreatic collection, mass lesion, or ascites is seen. Imaging features are characteristic of established chronic pancreatitis with ductal pathology.

Contrast-enhanced CT of the abdomen (Figure 1) demonstrated a diffusely atrophic pancreas with mild peripancreatic fat stranding and thickening of the left anterior pararenal fascia, interpreted as possible acute-on-chronic pancreatitis. A punctate calcification was noted in the pancreatic head, and the main pancreatic duct was markedly dilated (~9 mm) with abrupt distal narrowing at the ampullary region. No focal pancreatic mass, biliary obstruction, fluid collections, or surrounding bowel inflammation were identified.

Diagnostic integration

The coexistence of long-standing T1DM, autoimmune atrophic gastritis with pernicious anemia, extreme hypergastrinemia, autoimmune hypothyroidism, and severe exocrine pancreatic insufficiency with radiologic pancreatic atrophy, in the presence of preserved adrenal function, established a diagnosis of APS-3 with predominant gastrointestinal and pancreatic manifestations.

Taken together, the combination of long-standing T1DM, unexplained iron-restricted anemia, persistent vitamin B12 deficiency, marked hypergastrinemia, chronic gastrointestinal symptoms, and severe exocrine pancreatic insufficiency represented a constellation of clinical red flags suggestive of multisystem autoimmunity rather than isolated diabetic complications. This integrative recognition prompted formal classification as APS-3.

Management and clinical course

The patient was initiated on lifelong intramuscular vitamin B12 replacement, pancreatic enzyme replacement therapy, thyroid hormone replacement, and rationalization of acid-suppressive therapy. Over subsequent follow-up, he reported marked improvement in gastrointestinal symptoms, energy levels, and overall functional status.

Several days later, he experienced an episode of acute-on-chronic pancreatitis complicated by transient hepatocellular and cholestatic liver enzyme elevation and acute kidney injury. Imaging demonstrated dynamic biliary obstruction at the ampullary level, and endoscopic biliary decompression resulted in rapid biochemical recovery. At follow-up, renal function returned to baseline, liver enzymes normalized, inflammatory markers resolved, and the patient remained clinically stable on continued targeted therapy.

## Discussion

Autoimmune polyglandular syndrome type 3: epidemiology and clinical latency

APS-3 is defined by the coexistence of T1DM with autoimmune thyroid disease and/or autoimmune gastritis in the absence of adrenal insufficiency [2-4]. Large cohort studies indicate that 30-40% of individuals with T1DM develop at least one additional autoimmune disease during their lifetime, with autoimmune thyroid disease affecting approximately 20-30% and autoimmune gastritis reported in 10-30%, depending on screening strategy and disease duration [2,3]. Despite this prevalence, APS-3 remains frequently underrecognized in routine clinical practice due to its slow, asynchronous evolution and the absence of an early catastrophic organ failure that would prompt syndromic consideration [4].

**Figure 2:**
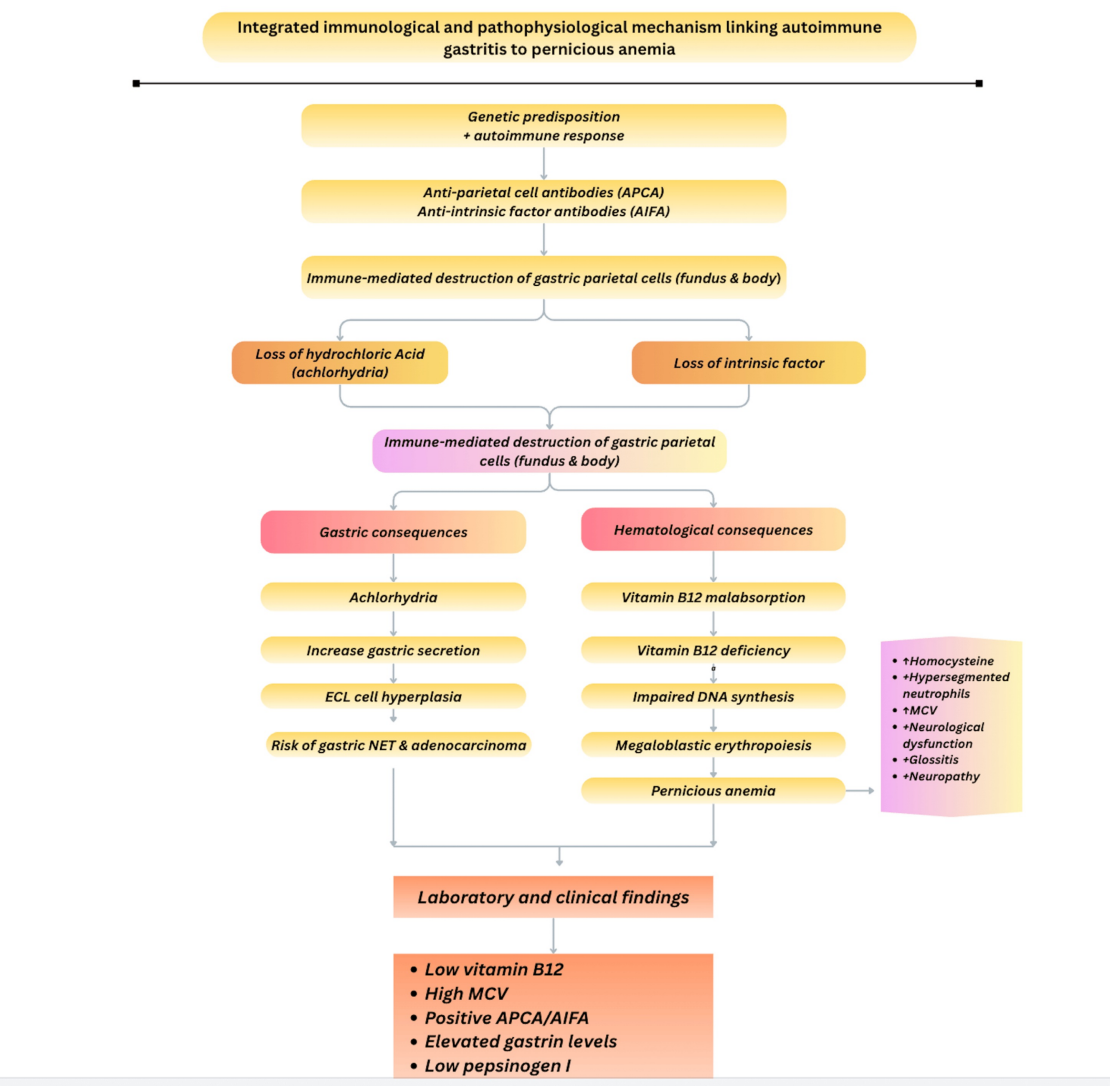
Integrated immunological and pathophysiological mechanism linking autoimmune gastritis to pernicious anemia. Integrated schematic illustrating the immunological and pathophysiological mechanisms linking autoimmune gastritis to pernicious anemia. Autoimmune destruction of gastric parietal cells leads to achlorhydria and intrinsic factor deficiency, resulting in vitamin B12 malabsorption, impaired DNA synthesis, and megaloblastic anemia. Laboratory and clinical correlations are shown. Illustration created by the authors using Canva.

Autoimmune gastritis follows a prolonged subclinical course. Longitudinal studies demonstrate that parietal cell antibodies may precede clinical diagnosis by more than a decade, during which progressive gastric atrophy develops silently [5]. Iron deficiency, often without macrocytosis, commonly precedes vitamin B12 depletion, reflecting early impairment of gastric acid-dependent iron absorption rather than late-stage intrinsic factor loss [5]. This temporal dissociation explains why autoimmune gastritis is often diagnosed only after neurological manifestations or overt pernicious anemia emerge, despite years of biochemical and immunologic warning signals. Figure 2 demonstrates the pathophysiological correlation between autoimmune gastritis and pernicious anemia.

In patients with T1DM, non-specific symptoms such as fatigue, anemia, and gastrointestinal discomfort are frequently attributed to diabetic complications, reinforcing diagnostic inertia [1,4]. APS-3 is therefore not rare from an epidemiologic standpoint, but systematically underdiagnosed due to fragmented, organ-centric clinical frameworks.

Pancreatic exocrine dysfunction in type 1 diabetes: beyond β-cell autoimmunity

Pancreatic involvement in T1DM has traditionally been conceptualized almost exclusively in terms of endocrine β-cell destruction [1]. However, accumulating evidence demonstrates that the exocrine pancreas is frequently affected both structurally and functionally. Reduced fecal elastase-1 levels have been reported in up to half of patients with long-standing T1DM, with a substantial subset exhibiting values consistent with severe exocrine pancreatic insufficiency [6]. Imaging studies corroborate these findings, demonstrating significant reductions in pancreatic volume independent of disease duration or glycemic control [7]. Histopathologic analyses further reveal immune-mediated inflammation, fibrosis, and acinar cell loss, supporting direct immune injury rather than secondary disuse or metabolic atrophy [8].

Despite these observations, exocrine pancreatic dysfunction in T1DM is often labeled subclinical, largely because overt steatorrhea is uncommon and symptom burden is inconsistently assessed [6-8]. This classification may underestimate its clinical relevance, particularly in patients with overlapping gastrointestinal autoimmunity. In such contexts, even partial impairment of digestive enzyme delivery can meaningfully exacerbate malabsorption, postprandial symptoms, and nutritional deficiency.

Interpreting fecal elastase in context

Fecal elastase-1 is a widely used non-invasive marker of exocrine pancreatic function, but its interpretation requires careful physiologic contextualization [9]. Mild-to-moderate reductions (100-200 µg/g) may occur in chronic diarrhea, hypochlorhydria, or rapid intestinal transit, reflecting dilutional effects rather than intrinsic pancreatic destruction [9]. In contrast, values below 100 µg/g, particularly when accompanied by weight loss, micronutrient deficiencies, or imaging abnormalities, strongly suggest clinically significant exocrine pancreatic insufficiency [9].

In autoimmune contexts, reliance on fecal elastase thresholds alone risks underestimating disease severity. In the present case, profoundly reduced elastase values in the single-digit range, diffuse pancreatic atrophy on imaging, exclusion of IgG4-related disease, and sustained symptomatic response to pancreatic enzyme replacement therapy collectively support true structural exocrine pancreatic failure rather than secondary functional hyposecretion [6-9].

Clinical implications: when to act on reduced fecal elastase

Importantly, the presence of reduced fecal elastase does not uniformly mandate pancreatic enzyme replacement therapy. Mild reductions are frequently encountered in patients with chronic gastrointestinal disorders, hypochlorhydria, or functional bowel disease and may represent secondary or reversible phenomena rather than irreversible pancreatic injury [9]. In such cases, empiric enzyme replacement may provide limited benefit and should be guided by symptom burden, nutritional markers, and overall clinical context rather than biochemical thresholds alone.

Conversely, fecal elastase values below 100 µg/g, particularly when associated with weight loss, fat-soluble vitamin deficiency, refractory gastrointestinal symptoms, or structural pancreatic abnormalities, strongly support clinically meaningful exocrine pancreatic insufficiency and justify therapeutic intervention [9]. In autoimmune disease, this distinction is especially important, as immune-mediated acinar injury may coexist with hypochlorhydria and malabsorption from autoimmune gastritis, compounding digestive inefficiency. In this patient, enzyme replacement therapy functioned not as empiric symptom control but as targeted correction of a previously unrecognized autoimmune-mediated digestive failure.

Autoimmune gastritis and hypergastrinemia as a central disease axis

Autoimmune atrophic gastritis represents a central driver of morbidity in APS-3. Immune-mediated destruction of gastric parietal cells leads to hypochlorhydria, impaired iron absorption, intrinsic factor deficiency, and vitamin B12 malabsorption [5]. Achlorhydria plays a pivotal upstream role in the clinical expression of pernicious anemia. Gastric acid is required for the liberation of protein-bound vitamin B12 from dietary sources; thus, progressive acid loss may initiate functional B12 insufficiency even before complete intrinsic factor depletion occurs. As parietal cell destruction advances, intrinsic factor deficiency supervenes, leading to true malabsorptive pernicious anemia.

**Figure 3:**
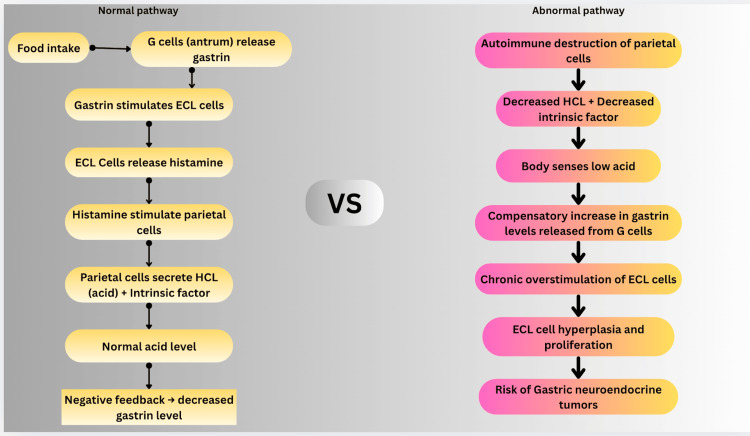
Normal versus abnormal gastric acid regulatory pathway. The diagram compares the normal control of gastric acid secretion with the abnormal pathway seen in disease. In normal physiology, acid production is tightly regulated by vagal stimulation, gastrin release, histamine action, and somatostatin inhibition. In pathological states, this balance is disturbed due to excessive stimulation and impaired inhibitory feedback, leading to hypochlorhydria/achlorhydria.This schematic flowchart was created by the authors to illustrate the mechanism described in this study. Illustration created by the authors using Canva.

Concomitant impairment of iron solubilization further complicates the hematologic picture, and mixed iron and B12 deficiency may produce normocytic indices that obscure classical macrocytosis. Extreme hypergastrinemia reflects advanced parietal cell loss and serves as a biologic marker of severe gastric secretory failure [10,11]. Gastrin levels exceeding 1,000 pg/mL indicate near-complete parietal cell destruction and sustained enterochromaffin-like cell stimulation, which has been associated with enterochromaffin-like cell hyperplasia and, in prolonged cases, type I gastric neuroendocrine tumor development [10,11]. Thus, achlorhydria not only precedes but modulates the clinical phenotype of vitamin B12 deficiency, contributing to delayed recognition and heterogeneous presentation. The pathophysiological cascade of autoimmune gastritis leading to hypergastrinemia and enterochromaffin-like cell hyperplasia is illustrated in Figure 3.

Dynamic biliary obstruction as a diagnostic confounder

Dynamic biliary obstruction further complicated the diagnostic course in this patient and illustrates an important clinical pitfall. Transient or fluctuating ampullary obstruction due to gallstones or biliary sludge is a well-described phenomenon and may produce marked hepatocellular enzyme elevations (“gallstone hepatitis”), often followed by spontaneous or partial biochemical improvement before definitive intervention [12]. Such dynamic patterns can mimic immune-mediated hepatitis and may be further modulated by anti-inflammatory therapies, leading to early but misleading improvements in aminotransferases [12]. In this case, definitive biliary decompression resulted in sustained biochemical recovery, confirming obstruction as the proximate trigger of the acute episode. Importantly, this episodic mechanical pathology occurred on a background of chronic autoimmune disease and pancreatic insufficiency, underscoring how acute reversible events may obscure but not negate underlying multisystem autoimmunity.

Immunogenetic and epithelial autoimmunity as a unifying framework

The coexistence of autoimmune gastritis, pancreatic exocrine insufficiency, and autoimmune thyroid disease in APS-3 suggests coordinated epithelial vulnerability rather than isolated endocrine failure [2-4]. These tissues share susceptibility to Th1-skewed immune responses characterized by interferon-γ signaling and aberrant antigen presentation [1,4]. Shared human leukocyte antigen (HLA) haplotypes, particularly HLA-DR3 and HLA-DR4, have been implicated across T1DM, autoimmune gastritis, and autoimmune thyroid disease, supporting a common immunogenetic substrate.

**Figure 4:**
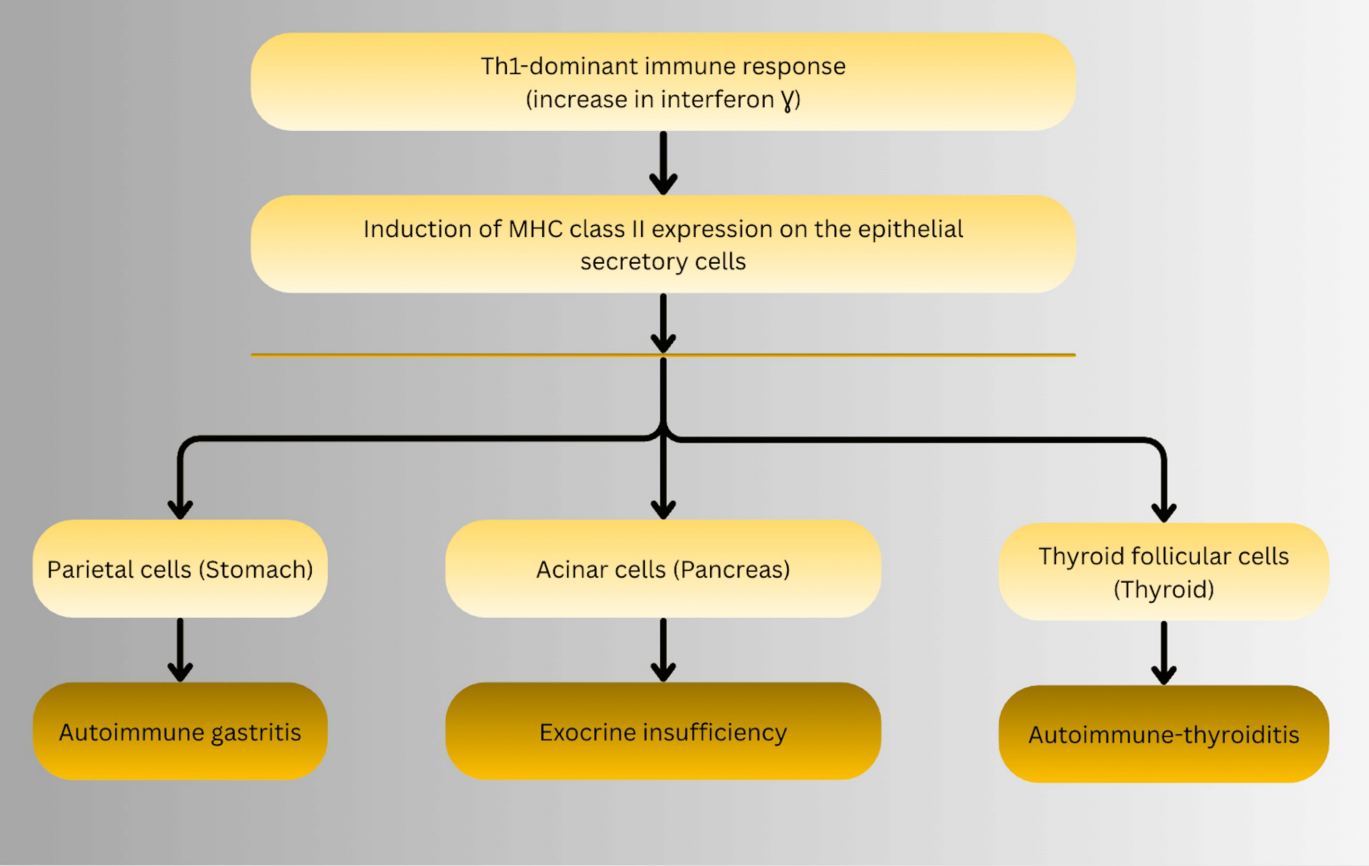
Proposed Th1-dominant immune mechanism linking epithelial major histocompatibility complex class II upregulation to multiorgan autoimmune injury. This mechanism is hypothesized to promote immune-mediated targeting of gastric parietal cells, pancreatic acinar cells, and thyroid follicular cells, contributing to autoimmune gastritis, exocrine insufficiency, and autoimmune thyroiditis. This model is based on current immunopathological understanding and ongoing investigation. Illustration created by the authors using Canva.

Experimental models demonstrate inducible major histocompatibility complex (MHC) class II expression on pancreatic acinar cells under inflammatory conditions, rendering them susceptible to immune-mediated injury [8]. Within this framework, pancreatic exocrine insufficiency in APS-3 is best understood as a collateral manifestation of systemic epithelial autoimmunity rather than a secondary metabolic complication. The extensive negative evaluation for alternative etiologies in this case, including exclusion of IgG4-related disease and autoimmune hepatitis, further strengthens the argument for a unified autoimmune pathogenesis rather than coincidental disease overlap. The proposed Th1-dominant immunopathogenic mechanism linking epithelial MHC class II upregulation to multiorgan autoimmunity is illustrated in Figure 4.

Histopathologic confirmation of autoimmune gastritis was not available for review, as prior endoscopy had been performed outside our institution, and documentation was inaccessible. However, the diagnosis was established based on strongly positive intrinsic factor and parietal cell antibodies, markedly elevated fasting gastrin levels consistent with corpus-predominant atrophy, and concordant biochemical evidence of pernicious anemia. Similarly, exocrine pancreatic insufficiency was supported by profoundly reduced fecal elastase values, radiologic pancreatic atrophy, exclusion of alternative etiologies, and sustained clinical response to pancreatic enzyme replacement therapy. In this context, the convergence of serologic, biochemical, radiologic, and therapeutic data provided sufficient diagnostic coherence without necessitating tissue confirmation.

Therefore, this case underscores that in multisystem autoimmune disease, diagnostic clarity often emerges from integration of converging clinical signals rather than reliance on isolated procedural endpoints. Recognition of APS-3 requires expansion beyond glycemic surveillance into symptom-driven autoimmune evaluation, particularly when persistent gastrointestinal and nutritional abnormalities coexist in long-standing T1DM.

## Conclusions

This case demonstrates that T1DM should be regarded as a sentinel autoimmune diagnosis rather than an isolated metabolic disorder. Failure to look beyond glycemic control can result in years of avoidable morbidity, misattributed symptoms, and delayed recognition of multisystem autoimmune disease. Recognition of APS-3 requires expansion of clinical suspicion beyond glycemic surveillance toward structured, symptom-driven autoimmune screening. Early identification of autoimmune gastritis, pancreatic exocrine insufficiency, and evolving endocrine involvement as components of APS enables targeted therapy, improves quality of life, and prevents prolonged patient suffering. Together, these observations underscore the need for a paradigm shift in endocrine practice toward integrated, symptom-driven autoimmune surveillance.
